# Electrical Storm in a Patient with Brugada Syndrome and Coronavirus Disease 2019

**DOI:** 10.19102/icrm.2022.130601

**Published:** 2022-06-15

**Authors:** Syed H. Ali, Kent R. Nilsson

**Affiliations:** ^1^Department of Medicine, Medical College of Georgia at the Augusta University–University of Georgia Medical Partnership, Athens, GA, USA; ^2^Department of Internal Medicine, Piedmont Heart Institute, Piedmont Athens Regional, Athens, GA, USA

**Keywords:** Brugada syndrome, COVID-19, fever, ventricular arrhythmia, ventricular storm

## Abstract

Brugada syndrome (BrS) is an inherited arrhythmia syndrome characterized by right bundle branch block and dynamic ST-segment changes in precordial leads V1–V3. In patients with BrS, fever is a known trigger that may induce arrhythmia. For patients with BrS who contract coronavirus disease 2019 (COVID-19), the inflammatory response poses the risk of causing ventricular arrhythmias. The following case discusses the management of a patient with BrS presenting with electrical storm after contracting COVID-19. Treatment should be focused on aggressive anti-pyretic management along with concomitant pharmacological therapy.

## Background

Brugada syndrome (BrS) is an inherited arrhythmia syndrome characterized by right bundle branch block and dynamic ST-segment changes in precordial leads V1–V3. One of the major causes of BrS is a mutation in the voltage-gated sodium channel, *SCN5A*, an ion channel whose conductance varies by alterations in temperature.^1,2^ As mutations that cause BrS reduce the sodium current, temperature fluctuations can further exacerbate alterations to the action potential, thereby increasing the risk of arrhythmias. In patients with BrS, fever is a known trigger that may induce arrhythmia.^3^

Coronavirus disease 2019 (COVID-19) is a coronavirus infection that was first identified in Wuhan, China in December 2019. The infection spread quickly worldwide, and the World Health Organization officially declared it a pandemic in March 2020.^4^ While the clinical manifestations of COVID-19 are legion, the severe inflammatory response to COVID-19 causes fever in the vast majority of symptomatic patients. To that end, levels of the cytokine interleukin-6, an inflammatory marker known to induce a febrile response, correlate with disease severity. For patients with BrS who contract COVID-19, the inflammatory response poses the additional risk of causing ventricular arrhythmias. The following case describes the management of a patient with known BrS presenting with electrical storm after contracting COVID-19.

## Case presentation

**Figure 1:**
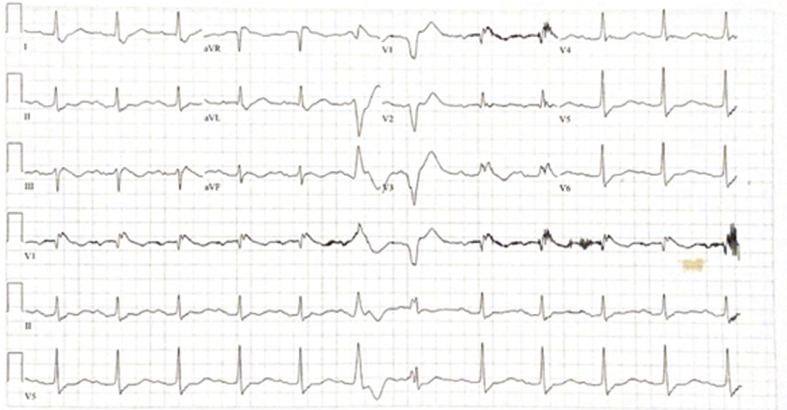
Electrocardiogram demonstrating the type 1 Brugada pattern.

A 73-year-old Caucasian man with a past medical history of BrS (documented *SCN5A* mutation), a history of electrical storm suppressed with quinidine, and an implantable cardioverter-defibrillator (ICD) in situ presented to the hospital with electrical storm. Earlier in the day, he had presented to an urgent care facility for dyspnea, dry cough, and a subjective fever for 1 week. He had mild nausea and loss of appetite. He was prescribed oral doxycycline and dexamethasone. Later in the day, he experienced multiple ICD discharges over the course of 1 hour, later confirmed on interrogation to be ventricular fibrillation (VF). Upon presentation, his vitals were stable, and the patient was afebrile. In the emergency room, the patient’s electrocardiogram **(Figure 1)** demonstrated a type 1 Brugada pattern. Quantitative reverse-transcription polymerase chain reaction (qRT-PCR) analysis confirmed COVID-19 infection. Chest X-ray showed bilateral peri-hilar opacities consistent with interstitial pneumonitis. Cardiology was consulted and recommended against β-blockers and amiodarone. Instead, he was started on acetaminophen, cilostazol, and quinidine. The patient was admitted to the floor and placed under modified enhanced respiratory and contact precautions for COVID-19.

**Figure 2:**
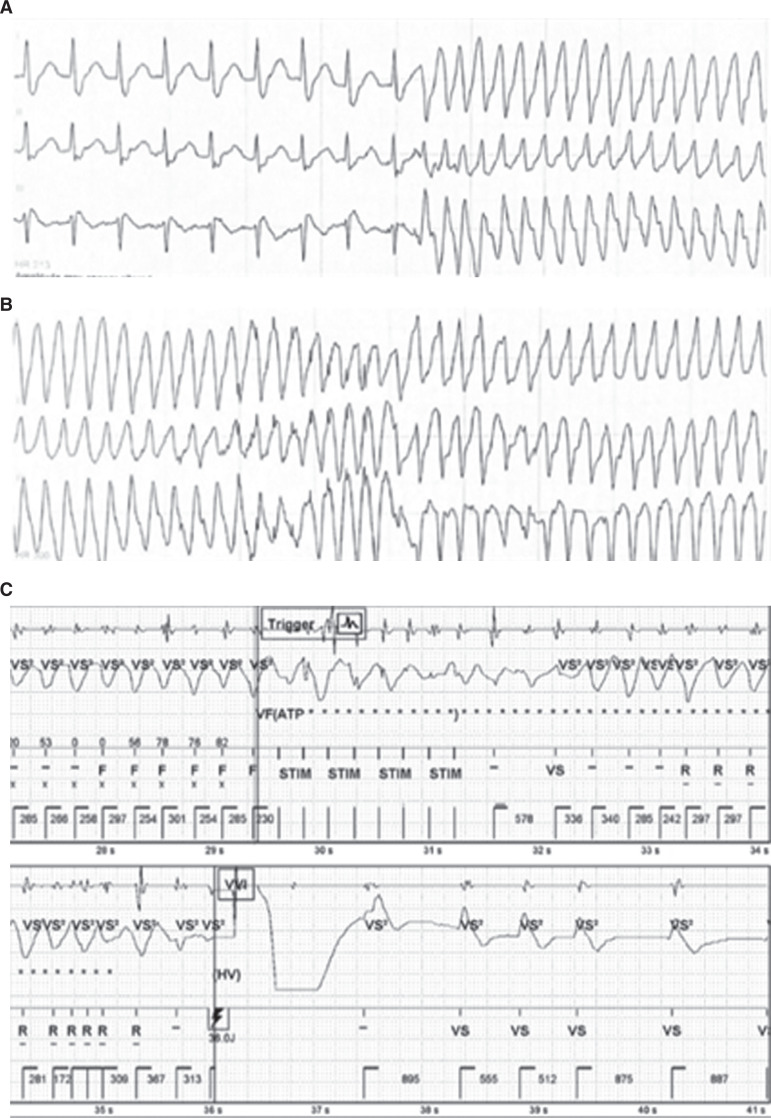
**A:** R-on-T initiation of polymorphic ventricular tachycardia. **B:** Attempt at anti-tachycardia pacing (ATP). **C:** Intracardiac electrocardiogram demonstrating ATP and defibrillation.

**Figure 3:**
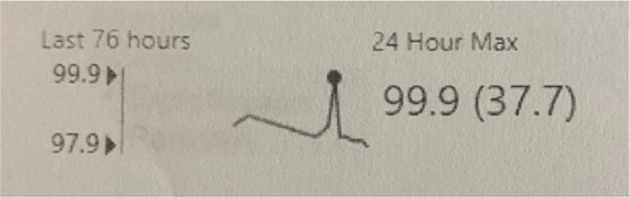
Temperature spike correlating with ventricular storm.

For his infection, he was given supportive care with bronchodilators and dexamethasone. He was also administered remdesivir and doxycycline for cough and placed on oxygen therapy for dyspnea. Overnight, the patient experienced electrical storm with 4 additional episodes of VF requiring defibrillation by his ICD within 10 min **(Figure 2)**. The onset correlated with a 1.5°F/0.8°C rise in his temperature up to 99.9°F/37.7°C **(Figure 3)**. He was given atropine and placed on an Isuprel^®^ drip (Bausch Health, Laval, Canada) to maintain a heart rate >90 bpm. Anti-pyretic therapy was augmented with the addition of ibuprofen. The patient was transferred to the intensive care unit for further monitoring.

Over the next 2 days, he did not experience recurrent VF, ventricular tachycardia (VT), or additional ICD discharges. Over the ensuing days, he defervesced and Isuprel^®^, followed by cilostazol, were discontinued. At discharge, metoprolol was discontinued and replaced with amlodipine for blood-pressure management. The patient was maintained on his outpatient prescription of quinidine. Eliquis^®^ (Bristol Myers Squibb, New York, NY, USA) was prescribed for anticoagulation prophylaxis secondary to COVID-19 infection.

## Discussion

BrS is seen characteristically with an ST-segment elevation in leads V1–V3 with a right bundle branch block pattern. It can wax and wane over time and may become pronounced during an acute illness, fever, drugs (flecainide, verapamil, propranolol), or during exercise. Three forms of BrS patterns have been described. Type 1 has a coved-type ST-segment elevation ;2 mm and a gradually descending ST segment followed by a negative T-wave. The type 1 Brugada pattern is the only accepted diagnostic pattern of BrS. It has a high predisposition for the onset of VT and further degeneration to VF and sudden cardiac death.^5^

### Impact of temperature on disease presentation

BrS is most commonly caused by a mutation in the *SCN5a* cardiac sodium channel. However, the majority of genetic abnormalities have yet to be identified. Interestingly, the ion channel conductance of sodium ions is disturbed at higher temperatures.^6^ Hence, if a patient with BrS is febrile, they are at high risk of developing an arrhythmia. COVID-19–induced fever is problematic for patients with BrS as it increases the risk of potentially fatal arrhythmias.

The aim for treatment of patients with BrS is to reduce the risk of sudden cardiac death secondary to polymorphic VT or VF.^7^ In our patient, a mild elevation in his temperature to 99.9°F/37.7°C led to repeated ICD discharges. Such a small increase in temperature is usually clinically irrelevant, but, in our patient, it led to electrical instability. Therefore, on admission, we recommend that patients should have temperatures monitored alongside telemetry to surveil for arrhythmogenesis.

Early and aggressive anti-pyretic therapy with Tylenol^®^ (Johnson & Johnson, New Brunswick, NJ, USA) plays a crucial role in stabilizing the cardiac sodium channel. In addition, ibuprofen has been shown to reduce cytokine interleukin-6 levels in previous studies via prostaglandin-mediated inhibition.^8,9^ Therefore, the prompt administration of ibuprofen will help reduce the cytokine storm and febrile response produced by COVID-19.

Regarding the non-pharmacological management of fever, external cooling may provide a potential benefit for BrS patients experiencing electrical storm. In a previous case study, therapeutic hypothermia was performed with a target temperature of 34°C in a patient with BrS who experienced an out-of-hospital cardiac arrest. The ST-segment elevation normalized and no aberrant ST-segment elevations in V1–V3 were noted. Clinical observation suggested that mild therapeutic hypothermia suppressed the Brugada pattern.^10^

### Pharmacologic options to suppress electrical storm in Brugada syndrome

Pharmacologic therapy targeting the membrane action potential can also help prevent arrhythmogenesis. Quinidine, a class 1A anti-arrhythmic drug, is the most extensively used drug for the prevention of arrhythmias in BrS patients.^11^ Quinidine has been shown to normalize the epicardial action potential dome and the ST segment. It has prevented polymorphic VT in experimental studies by blocking the phase I I_to_ current, thereby compensating for the reduced influx of sodium during phase 0.^12^

Cilostazol is an oral phosphodiesterase inhibitor that is mainly used for its antiplatelet and/or vasodilatory properties. In the setting of BrS, a previous case study reported its use in preventing VF. This phenomenon was confirmed by an “on-and-off” challenge, where discontinuation of the drug resulted in VF and re-administration halted VF. Two proposed mechanisms of action were put forward, as follows: (1) suppression of the I_to_ channel secondary to an increase in heart rate and (2) an increase in Ca^2+^ current secondary to increased intracellular cyclic adenosine monophosphate via the inhibition of phosphodiesterase activity.^13^ Given the heterogeneous nature of disease-causing mutations in BrS, the salutary benefits of cilostazol may be limited to a subset of patients.

In patients with BrS, β-blockers are known to reduce the inward Ca^2+^ current and cause an outward shift, creating a transmural voltage gradient, leading to ST-segment elevation and the potentiation of VF.^14^ Therefore, it is imperative to discontinue β-blockers in these patients. Following discontinuation, the immediate administration of Isuprel^®^, a β-adrenergic agonist, is recommended to suppress VF. It restores the action potential dome by increasing the influx of Ca^2+^ secondary to an elevation of intracellular cyclic adenosine monophosphate.^15^ In our patient, there was no recurrent VF or VT following administration of Isuprel^®^ and suppression of fever.

## Conclusion

Symptomatic COVID-19 with fever can cause electrical storm in patients with BrS. Electrocardiographic monitoring of COVID-19 patients should be recommended to identify life-threatening arrhythmias during periods of fever and cytokine storm. Treatment for fever-induced BrS should be focused on aggressive anti-pyretic management with concomitant pharmacological therapy.
